# Association of preoperative CA-125 levels with early POAF after heart valve surgery: a single-center, retrospective study

**DOI:** 10.1186/s12893-023-02099-z

**Published:** 2023-08-09

**Authors:** Xiaoqin Liao, Sailan Li, Xin Yan, Xin Lin, Liangwan Chen, Yanjuan Lin

**Affiliations:** 1 Department of Cardiovascular Surgery, Fujian Medical University Union Hospital, Fuzhou, China; 2 Department of Nursing, Fujian Medical University Union Hospital, Fuzhou, China

**Keywords:** Postoperative atrial fibrillation, Cancer antigen-125, Cardiac surgery, Valve surgery

## Abstract

**Objective:**

Cancer antigen-125 (CA-125), a tumor marker, has received increasing attention in recent years for its role in the cardiovascular field. However, no study has reported the association of CA-125 with early postoperative atrial fibrillation (POAF) after heart valve surgery. Therefore, the aim of this study was to assess whether there is a correlation between CA-125 and early postoperative POAF after heart valve surgery.

**Methods:**

Patients who underwent valve surgery at Fujian Heart Medical Center from January 2020 to August 2022 were retrospectively analyzed and divided into postoperative atrial fibrillation group (POAF group) and postoperative non-atrial fibrillation group (NO-POAF), and the differences in clinical data between the two groups were compared, and the variables with statistical significance in the univariate analysis were included in the COX regression analysis, and finally the receivers’ operating characteristics (ROC) curves were drawn.

**Results:**

From January 2020 to August 2022, a total of 1653 patients underwent valve surgery. A total of 344 patients were finally included, including 52 patients (15.1%) in the POAF group and 292 patients (84.9%) in the NO-POAF group. Univariate analysis showed higher CA-125 levels in patients in the POAF group than in those in the NO-POAF group [27.89 (13.64, 61.54), 14.48 (9.87, 24.08), P = 0.000]. Analysis of the incidence of POAF based on CA-125 quartiles showed an incidence of up to 29.2% in the highest quartile (> 27.88). Multivariate COX regression analysis showed that CA-125 [OR = 1.006, 95% CI (1.002, 1.010), P = 0.001] was an independent predictor of POAF. The final ROC curve plot showed that the area under the curve for CA-125 was 0.669, with an optimal cut-off value of 27.08 U/ml, and the difference in the area under the curve between the two groups was statistically significant (P = 0.000).

**Conclusion:**

Elevated preoperative CA-125 levels can affect the incidence of POAF and have a predictive value for the occurrence of POAF in the early stage after valve surgery. However, due to the small sample size and single-center retrospective study, further validation of this result is needed.

## Introduction

Atrial fibrillation (AF) is one of the most common arrhythmias with a high morbidity and mortality risk [1]. And the incidence of AF in postoperative cardiac patients can be 20-62% [2–4]. Postoperative atrial fibrillation (POAF) usually occurs during a patient’s hospitalization, has a short duration, and rarely develops into a chronic disease, but it can prolong hospitalization, increase medical costs, and patient mortality [5, 6]. However, there are relatively few studies on the occurrence of POAF in patients undergoing cardiac surgery, and there are insufficient and incomplete studies. Therefore, it is necessary to further investigate the risk factors for the occurrence of POAF in patients undergoing cardiac surgery in order to promote disease recovery and reduce mortality [7, 8].

Cancer antigen-125 (CA-125) is a macromolecular soluble protein, usually produced by luminal endothelial cells (e.g., pleura, peritoneum, and pericardium), and is a biomarker for monitoring the efficacy of ovarian cancer treatment [9]. In recent years, many studies have reported elevated CA-125 levels in patients with cardiac disease. Nagele et al. [10] first reported elevated CA-125 levels after heart transplantation. Recently, Pandhi et al. [11] reported elevated CA-125 levels in patients with heart failure, Sekiguchi et al. [12] suggested that high CA-125 levels were an independent predictor of new-onset AF in healthy postmenopausal women, and Wang et al. [13] suggested that elevated preoperative CA-125 levels were associated with an increased risk of AF recurrence and independently predicted recurrence of AF after radiofrequency catheter ablation (RFCA).

Although studies have reported that CA-125 levels are elevated in patients with heart disease and correlate with disease prognosis, few studies have reported the relationship between POAF and CA-125 in the early postoperative period after heart valve surgery. Therefore, the aim of this study was to investigate the correlation between preoperative CA-125 levels and POAF in the early postoperative period after heart valve surgery.

## Methods

### Study population

The study population was patients who underwent heart valve surgery at Fujian Heart Medical Center from January 2020 to August 2022. All patients were ≥ 18 years of age, free of preoperative atrial fibrillation, metabolic disease, malignancy, history of autoimmune disease, and severe liver and kidney insufficiency. Patients with a history of cardiac disease, treated with steroids and/or immunosuppressive drugs were excluded. The need for written informed consent was waived as this study was based on routine clinical data for retrospective analysis.

### Data collection

Relevant clinical datas of the patients were collected from the hospital medical record system, including (1) general clinical data: gender, age, body mass index (BMI), smoke, drink, hypertension, and diabetes mellitus; (2) preoperative clinical data: cardiac function classification (NYHA cardiac function classification classifies the impaired status of cardiac function into class I to IV according to the activity of the induced heart failure symptoms), left ventricular ejection fraction (LVEF), leukocytes count, neutrophil count, erythrocyte count, platelet count, hemoglobin, carcinoembryonic antigen (CEA) level, Cancer antigen-125 level, Cancer antigen-199 (CA-199) level; (3) intraoperative clinical data: operation time, extracorporeal circulation time; (4) postoperative clinical data. Intensive care unit hospitalization days, duration of mechanical ventilation, cardiac output (CO).

### Assessment of atrial fibrillation

Patients were admitted to the ICU for vital signs monitoring after surgery, and the patients were judged to be in atrial fibrillation according to the data displayed by the cardiac monitoring instruments [14]. POAF was diagnosed if the patient had no preoperative atrial fibrillation and was monitored for the occurrence of atrial fibrillation after surgery; atrial fibrillation occurring from the day of surgery to 7 days after surgery was considered early postoperative POAF.

### Statistical analysis

All statistical analyses were completed using SPSS software version 23.0. Continuous variables that conformed to a normal distribution were expressed as mean ± standard deviation (SD) using the t test. And variables that did not conform to a normal distribution were expressed as medians (quartiles) using the Wilcoxon signed rank sum test. Categorical data were then expressed using the number of cases (%) and the chi-square or Fisher exact test was used to compare categorical data. A multistep process was used to investigate the effect of CA-125 levels on POAF in the early postoperative period after heart valve surgery. First, patients were divided into four strata for analysis based on quartiles of CA-125 levels. Second, statistically significant variables of interest identified in the univariate analysis were entered into multivariate COX regression analysis, and finally, the area under the curve (AUC) was calculated by subject operating characteristic (ROC) curve analysis. A two-sided P < 0.05 was considered a statistically significant difference.

## Results

**Fig. 1:**
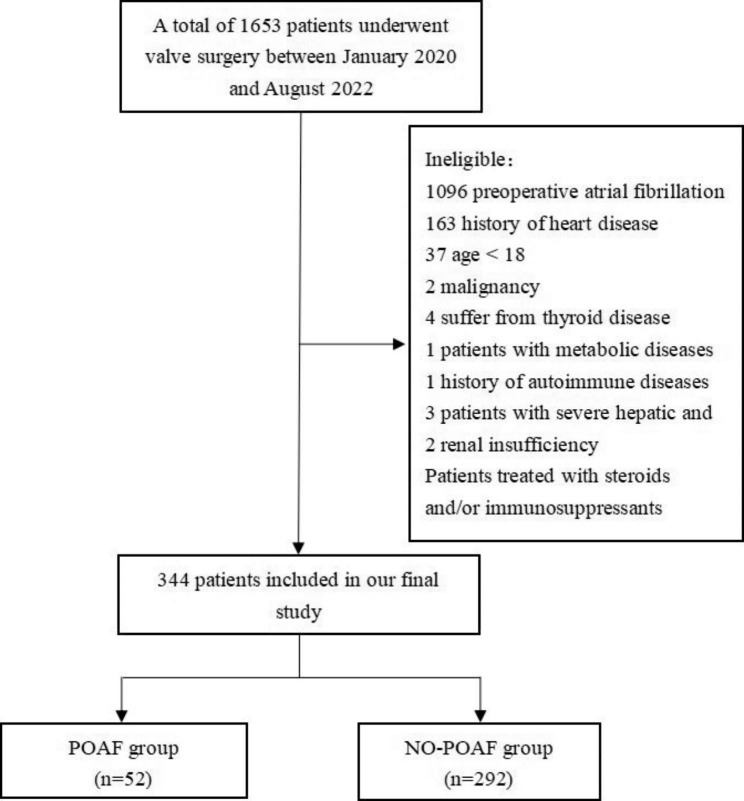
Flowchart of patient inclusion

From January 2020 to August 2022, a total of 1653 patients underwent valve surgery. A total of 1309 patients (including 1096 patients with preoperative atrial fibrillation, 163 patients with a history of cardiac disease, 37 patients younger than 18 years, 2 patients with malignancy, 4 patients with thyroid disease, 1 patient with metabolic disease, 1 patient with a history of autoimmune disease, 3 patients with severe liver and kidney insufficiency, and 2 patients treated with steroids and/or immunosuppressive drugs) were excluded, and finally a total of 344 patients were included, including 52 patients in the POAF group and 292 patients in the NO-POAF group. The flow chart of patient inclusion is shown in Fig. 1.

**Fig. 2:**
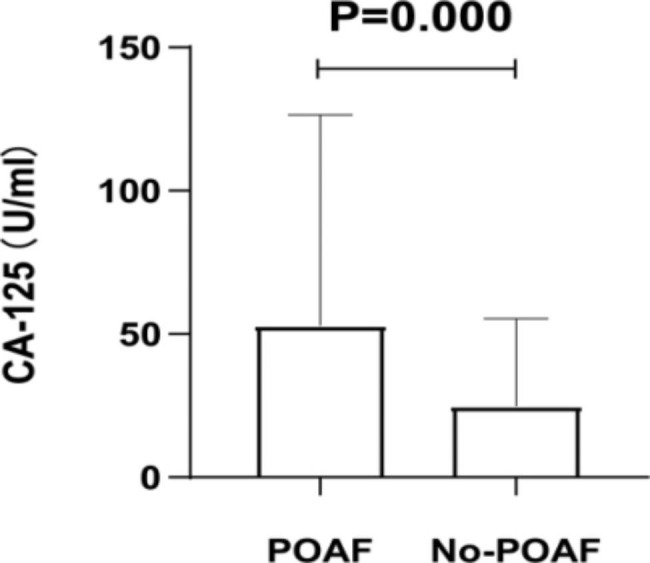
Box-plot of preoperative CA-125 levels in the POAF and NO-POAF groups

Table 1 shows the baseline and clinical characteristics of patients in the POAF and NO-POAF groups. 52 (15.1%) of 344 patients were in the POAF group and 292 (84.9%) were in the NO-POAF group. The POAF and NO-POAF groups in terms of the percentage of females (63.5% vs. 45.9%), age (62.00 ± 9.21 vs. 58.578 ± 11.17), prevalence of hypertension (63.5% vs. 44.2%), leukocyte level (13.32 ± 3.78 vs. 11.85 ± 3.84), days of intensive care unit stay [2.20 (1.33, 3.80) vs. 2.10 (1.40, 3.00)] were statistically significant different (p<0.05). CA-125 levels [27.89 (13.64, 61.54) vs. 14.48 (9.87, 24.08), P = 0.000] were even more statistically significantly different (P < 0.01) for comparison (see Fig. 2). The rest of the variables were not statistically different for comparison (P > 0.05).

**Table 1:** Baseline patient characteristics

Variables	POAF Group (n = 52)	NO-POAF Group (n = 292)	Test Statistics	*P-*Value
General information of patients				
Gender			5.456	0.020
Male, n(%)	19(36.5)	158(54.1)		
Female, n(%)	33(63.5)	134(45.9)		
Age	62.00 ± 9.21	58.578 ± 11.17	32.093	0.037
BMI	23.45 ± 3.62	23.11 ± 3.55	0.629	0.530
Smoke			0.467	0.494
Yes, n(%)	7(13.5)	30(10.3)		
No, n(%)	45(86.5)	262(89.7)		
Drink			0.471	0.493
Yes, n(%)	9(17.3)	40(13.7)		
No, n(%)	43(82.7)	252(86.3)		
Hypertension			6.588	0.010
Yes, n(%)	33(63.5)	129(44.2)		
No, n(%)	19(36.5)	163(55.8)		
Diabetes			2.324	0.127
Yes, n(%)	22(44.3)	92(31.5)		
No, n(%)	30(57.7)	200(68.5)		
Pre-operative information of patients				
Cardiac function classification			5.965	0.051
Class I	0(0)	0(0)		
Class II	33(63.5)	229(78.4)		
Class III	17(32.7)	69(23.6)		
Class IV	2(3.8)	4(1.4)		
LVEF(%)	56.50(52.00, 61.75)	57.00(55.00, 62.00)	1.371	0.170
Leukocyte 10^9^/L	13.32 ± 3.78	11.85 ± 3.84	2.541	0.011
Neutrophil 10^9^/L	9.80(8.50, 12.08)	9.65(8.25, 10.77)	1.212	0.226
Erythrocyte10^12^/L	4.46 ± 0.61	4.51 ± 0.78	0.500	0.617
Blood platelet 10^9^/L	161.00(128.00, 236.5)	172.50(142.00, 235.00)	0.837	0.403
Hemoglobin g/L	131.25 ± 13.53	133.10 ± 13.25	0.930	0.353
CEA U/ml	2.20(1.33, 3.80)	2.10(1.40, 3.00)	1.033	0.301
CA-125 U/ml	27.89(13.64, 61.54)	14.48(9.87, 24.08)	3.889	0.000
CA-199 U/ml	12.33 ± 6.63	11.82 ± 8.91	0.390	0.697
Intraoperative information of patients				
Operation time (min)	273.70 ± 67.30	261.03 ± 68.52	1.189	0.235
Extracorporeal circulation time (min)	124.17 ± 32.68	116.71 ± 31.22	1.576	0.116
Post-operative information of patients				
Days of ICU stay(d)	2.20(1.33, 3.80)	2.10(1.40, 3.00)	2.691	0.007
Duration of mechanical ventilation(h)	19.00(16.00, 22.00)	18.00(15.00, 21.00)	1.149	0.251
CO L/min	5.40(4.10, 7.20)	5.8(4.60, 7.78)	1.067	0.287

Note: BMI: Body Mass Index; LVEF: Left ventricular ejection fraction; CEA: Carcinoembryonic antigen; CA-125: Glyco-antigens − 125; CA-199: Glyco-antigens − 199; CO: Cardiac Output

Table 2 Comparison of patients’ clinical data according to CA-125 quartiles. The results showed significant differences in CA-125 quartiles, cardiac function classification, leukocytel count, CEA level, and days of intensive care unit stay (P < 0.05).

**Table 2:** Comparison of clinical profiles of patients in the CA-125 quartiles

Variables	CA125					Statistical values	*P*-Value
	Quartile 1 (n = 86)	Quartile 2 (n = 87)		Quartile 3 (n = 82)	Quartile 4 (n = 89)		
**General information of patients**							
Gender							
Male, n(%)	45(52.3%)		44(50.6%)	37(45.1%)	51(57.3%)	2.588	0.460
Female, n(%)	41(47.7%)		43(49.4%)	45(54.9%)	38(42.7%)		
Age	58.76 ± 10.68		58.21 ± 11.29	59.13 ± 12.33	60.21 ± 9.55	0.525	0.665
BMI	22.42(20.50, 24.96)		22.89(20.70, 25.26)	21.76(20.50, 25.60)	23.40(20.90, 25.65)	2.465	0.482
Smoke						5.826	0.120
Yes, n(%)	4(4.7)		9(10.3)	10(12.2)	14(15.7)		
No, n(%)	82(95.3)		78(89.7)	72(87.8)	75(84.3)		
Drink						2.046	0.563
Yes, n(%)	9(10.5)		12(13.8)	12(14.6)	16(18)		
No, n(%)	77(89.5)		75(86.2)	70(85.4)	73(82)		
Hypertension						0.356	0.949
Yes, n(%)	42(48.8)		39(44.8)	38(46.3)	43(48.3)		
No, n(%)	44(51.2)		48(55.2)	44(53.7)	46(51.7)		
Diabetes						3.682	0.298
Yes, n(%)	22(25.6)		28(32.2)	31(37.8)	33(37.1)		
No, n(%)	64(74.4)		59(67.8)	51(62.2)	56(62.9)		
**Pre-operative information of patients**							
Cardiac function classification						13.692	0.015
Class I	0		0	0	0		
Class II	72		72	61	57		
Class III	14		14	18	30		
Class IV	0		1	3	2		
LVEF(%)	63.70(56.80, 68.90)		64.60(57.80, 69.2)	62.9(54.5, 68.85)	63.00(57.35, 68.9)	0.675	0.879
Leukocyte 10^9^/L	9.88(8.70, 11.64)		10.67(8.93, 14.35)	11.81(8.34, 14.18)	11.42(8.99, 15.90)	11.95	0.008
Neutrophil 10^9^/L	9.48(8.14, 11.01)		9.62(8.23, 10.98)	9.75(8.57, 10.98)	9.74(8.30, 10.63)	0.727	0.867
Lymphocyte 10^9^/L	6.64 ± 1.58		6.47 ± 1.63	6.84 ± 1.54	6.98 ± 1.74	1.689	0.169
Erythrocyte 10^12^/L	4.50 ± 0.79		4.46 ± 0.74	4.50 ± 0.80	4.56 ± 0.73	0.274	0.844
Blood platelet 10^9^/L	174.00(137.75, 235.50)		169.00(143.00, 235.00)	165.5(137.00, 221.75)	169.00(139.00, 236.00)	0.744	0.863
Hemoglobin g/L	133.00(125.75, 139.75)		132.00(125.00, 142.00)	135.00(126.00, 144.50)	134.00(125.00, 142.50)	0.667	0.573
CEA U/ml	2.00(1.40, 2.60)		1.90(1.10, 2.60)	2.20(1.50, 3.03)	2.70(1.55, 4.05)	15.939	0.001
CA-199 U/ml	9.28(5.86, 14.39)		10.78(6.79, 15.92)	10.15(6.83, 14.48)	9.71(6.32, 14.81)	1.469	0.689
**Intraoperative information of patients**							
Operation time (min)	237(214.5, 280.25)		243(215, 301)	243.5(216, 297.25)	253(235.5, 313.5)	5.954	0.114
Extracorporeal circulation time (min)	113.15 ± 30.50		114.70 ± 30.21	118.96 ± 32.03	122.85 ± 29.53	1.797	0.147
**Post-operative information of patients**							
Days of ICU stay(d)	2.00(2.00, 3.00)		2.00(2.00, 3.00)	2.00(2.00, 3.00)	3.00(2.00, 3.00)	8.103	0.044
Duration of mechanical ventilation(h)	18.00(15.00, 21.00)		18.00(15.00, 21.00)	18.00(15.00, 21.00)	19.00(15.50, 23.00)	4.067	0.254
CO L/min	6.50(4.90, 7.93)		6.00(4.80, 7.30)	5.55(4.18, 7.15)	5.30(4.20, 7.20)	7.731	0.052

Table 3 shows the incidence of early POAF in patients undergoing valve surgery based on CA-125 quartiles. The results show a significant difference in the incidence of POAF in the CA-125 quartiles (P < 0.05), with an incidence of up to 29.2% in the highest quartile (> 27.88).

**Table 3:** Incidence of POAF in patients undergoing valve surgery based on CA-125 quartiles

Variables	The level of CA125				*P*-Value
	Quartile 1	Quartile 2	Quartile 3	Quartile 4	
Quartile values U/ml	<10.08	10.08–15.43	15.44–27.88	>27.88	
POAF/NO-POAF	8/78	9/78	9/73	26/63	
Morbidity(%)	9.3%	10.3%	11%	29.2%	0.000

### COX regression analysis of possible predictors of POAF

The variables that were statistically significant in the univariate analysis were included in the multivariate COX regression analysis, which showed that CA-125 [OR = 1.006, 95% CI (1.002, 1.010), P = 0.001] was an independent predictor of POAF. See Table 4.

**Table 4:** POAF multivariate COX regression analysis

Variables	B	SE	Wald value	*P*	OR	95%CI	
						Lowest limit	highest limit
Age	-0.023	0.013	3.113	0.078	0.977	0.953	1.003
Gender	0.064	0.287	0.050	0.823	1.066	0.608	1.871
Hypertension	-0.473	0.303	2.440	0.118	0.623	0.344	1.128
Leukocyte 10^9^/L	0.029	0.034	0.723	0.395	1.030	0.963	1.102
CA-125 U/ml	0.006	0.002	10.552	0.001	1.006	1.002	1.010

### ROC graph

**Fig. 3:**
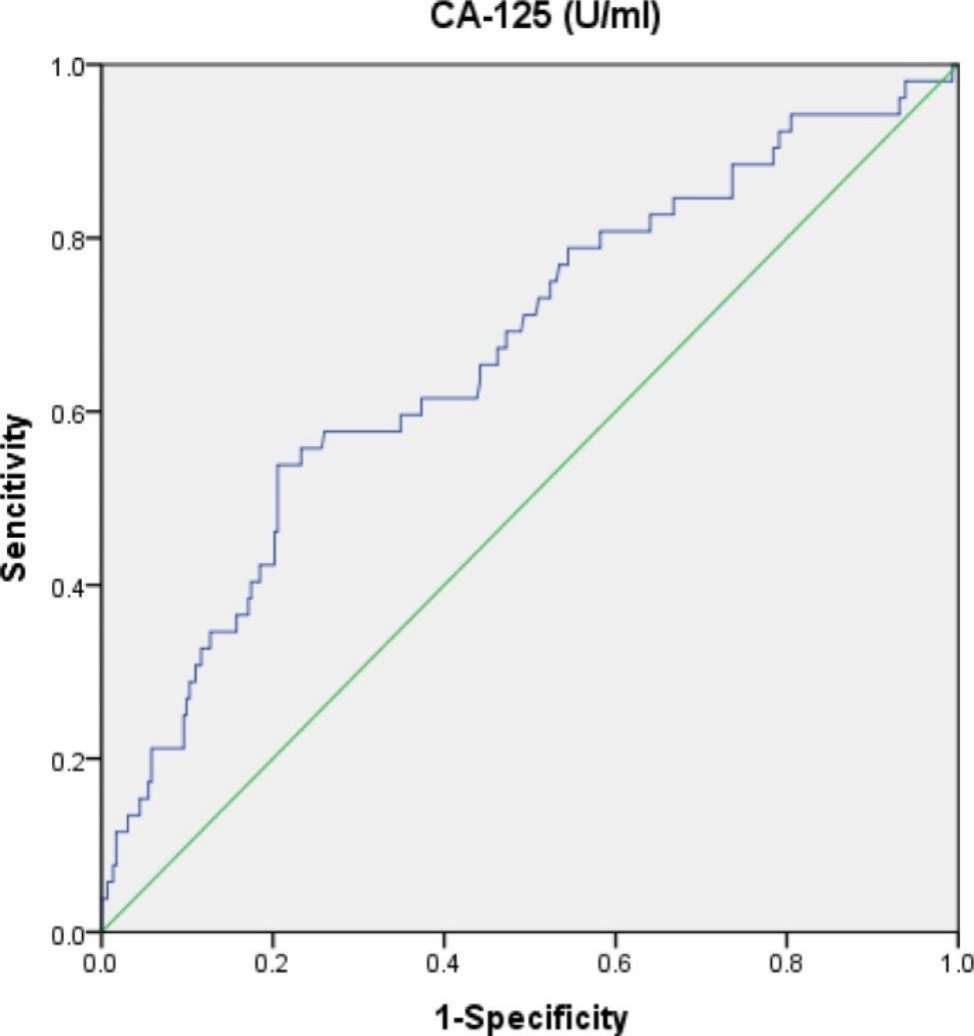
CA-125 ROC graph

According to the ROC curve plot in Fig. 3, the area under the curve of CA-125 was 0.669 (P = 0.000), the best cut-off value was 27.08 U/ml, the Yordon index was 0.333, the sensitivity was 53.8% and the specificity was 79.5%. The difference in the area under the curve between the two groups was statistically significant.

## Discussion

The dangers of POAF are well known, although we have taken many approaches to try to reduce its incidence, our findings show that the incidence of POAF, at 15.1%, is still at a high level. Therefore, we need to understand more deeply the risk factors that cause the occurrence of POAF in order to take targeted measures to reduce the occurrence of POAF.

At present, the study of CA-125 in the cardiovascular field is gradually deepening, and our findings show that preoperative CA-125 levels are associated with the occurrence of POAF early after valve surgery. Other scholars have also concluded that CA-125 levels are correlated with cardiovascular disease and its prognosis. Cheung et al. [15] derived results from systematic evaluation and meta-analysis showing that CA-125 levels were higher in patients with AF than in patients with sinus rhythm and that elevated CA-125 levels were associated with an increased risk of AF, but there was a high problem of heterogeneity between included studies, so this conclusions may be subject to some uncertainty.Núñez et al. [16] concluded that CA-125 levels predicted 1-year mortality and readmission rates in patients with heart failure.Eggers et al. [17] concluded that CA-125 was correlated with all-cause mortality in patients with acute myocardial infarction by analyzing 175 biomarkers.

The physiological role of CA-125 is to moisturize and lubricate the epithelial luminal surfaces and protect them from mechanical stress and stretching applied to the cells [18]. In contrast, the potential mechanisms of elevated CA-125 levels in cardiovascular disease, including atrial fibrillation, are multifaceted. Impaired ventricular function, left atrial remodeling, vascular mechanics and increased oxidative stress may induce CA-125 release from epithelial luminal cells of plasma membrane tissue [19]. In addition, elevated inflammatory biomarkers (e.g., interleukin-6, C-reactive protein, and tumor necrosis factor-α) can promote CA-125 secretion to some extent [20, 21].

In ovarian cancer detection, a CA-125 serum value < 35 U/mL is usually considered normal [9]. In contrast, Sekiguchi et al. [12], by analyzing new-onset AF in postmenopausal women, showed that the optimal critical level of CA-125 for predicting new-onset AF was ≥ 9.8 U/mL. Yucel et al. [22] reported that the optimal critical level of CA-125 for predicting the development of AF in patients with systolic chronic heart failure was ≥ 68.49 U / mL. Our study showed by analysis that the most predictive value of early POAF in postvalvular patients was 27.08 U/ml. From the current findings, CA-125 has a certain influence on cardiovascular diseases and their prognosis, but there is variability in the findings, and we need to conduct more in-depth studies.

### Limitations of this study

There are some limitations of this study. (1) The sample size of this study is small and it is a single-center retrospective study. (2) We only included CA-125 levels measured preoperatively in patients and did not assess the effect of postoperative CA-125 levels on cardiovascular disease or whether postoperative CA-125 changed over time. (3) In this study, we only analyzed the correlation between preoperative CA-125 levels and early POAF in patients after valve surgery, and did not statistically analyze the occurrence of distant POAF and the factors influencing it.

## Conclusion

The results of this study showed a correlation between CA-125 levels and the occurrence of early POAF after heart valve surgery, but further validation of this result is needed subsequently because this study is a small sample, single-center retrospective study.

## Data Availability

The raw data set used to support the results of this study is available upon request from the corresponding author(Lin Yanjuan).
